# *Rhododendron luohanbaense* (Ericaceae), a new species from northeast Yunnan, China

**DOI:** 10.3897/phytokeys.273.182747

**Published:** 2026-04-23

**Authors:** Yong-Li Zhao, Chao-Shan Gu, Zheng-Hang Liao, Jing-Li Zhang, Wei Li, Zhu-Fang Xie, Fei Wang, Yong-Peng Ma, Xing-Xing Mao, De-Tuan Liu

**Affiliations:** 1 Administration Bureau of Yunnan Wumengshan National Nature Reserve, Zhaotong 657099, Yunnan, China Zhejiang Normal University Jinhua China; 2 College of Horticulture and Landscape, Yunnan Agricultural University, Kunming 650201, Yunnan, China Kunming Institute of Botany, Chinese Academy of Sciences Kunming China; 3 Key Laboratory of Ecology of Rare and Endangered Species and Environmental Protection, Guangxi Normal University, Guilin 541000, Guangxi China Guangxi Normal University Guilin China; 4 West China Subalpine Botanical Garden, Institute of Botany, Dujiangyan 611834, Sichuan, China Yunnan Agricultural University Kunming China; 5 Yunnan Key Laboratory for Integrative Conservation of Plant Species with Extremely Small Populations, Kunming Institute of Botany, Chinese Academy of Sciences, Kunming 650201, Yunnan, China Administration Bureau of Yunnan Wumengshan National Nature Reserve Zhaotong China; 6 College of Life Sciences, Zhejiang Normal University, Jinhua 321004, Zhejiang, China West China Subalpine Botanical Garden, Institute of Botany Dujiangyan China

**Keywords:** Ericaceae, new taxon, nuclear orthologous genes, phylogeny

## Abstract

*Rhododendron luohanbaense*, a new species of Ericaceae, is described from Zhaotong City in northeastern Yunnan Province, China. Morphologically, it is most similar to both *R. pachytrichum* and *R. maculiferum* and can be classified into the subgen. *Hymenanthes* sect. *Pontica* subsect. *Maculifera*. However, it can be easily distinguished by markedly smaller and narrower leaves with acuminate apex, abaxial midribs densely covered with woolly indumentum, yellowish-green corollas speckled with green or purple dots, inflorescences bearing up to 7 flowers, flowers with ovary densely pilose, capsule densely brown setose. Phylogenomic evidence, based on a coalescent-based species tree reconstructed from 5358 single-copy nuclear orthologous genes, also supports recognition of the new taxon.

## Introduction

The genus *Rhododendron*, first described by Linnaeus (1753: 392), comprises more than 1000 species worldwide. China exhibits a remarkable diversity, with more than 600 species documented (Geng 2014). Even after the publication of the Flora of China (Fang et al. 2005), new species of *Rhododendron* continue to be discovered and reported from China in recent years (Tian et al. 2019; Deng et al. 2025; Zhao et al. 2025).

The southwestern region of China, particularly the Provinces of Yunnan, Sichuan, Xizang, and Guizhou, represents a major center of distribution and diversity for this genus. The Wumengshan National Nature Reserve is located in northeastern Yunnan Province, spanning a critical floristic ecotone at the convergence of the Yunnan-Guizhou Plateau and the Sichuan Basin. Owing to its distinctive geographic setting, complex topography, and diverse altitudinal climates, the reserve harbors a wide array of vegetation types, intricate floristic compositions, and rich biodiversity (Zhao et al. 2024). More than 2000 species of vascular plants have been recorded within the reserve. The montane climate of the reserve, characterized by relatively low mean annual temperatures (6–15 °C) and high relative humidity (>85%), creates cool mesic environments and ecological niches that are highly suitable for rhododendrons.

Three new species in the genus *Rhododendron* were discovered during a series of field surveys conducted in the nature reserve over the years, i.e. *R. rimicola* X.L. Tian, Y.P. Ma & J. Nielsen (Tian et al. 2019), *R. kuomeianum* Y.H. Chang, J. Nielsen & Y.P. Ma (Chang et al. 2021) and *R. wumengense* Y.L. Zhao & D.T. Liu (Zhao et al. 2025). Subsequent surveys revealed a further taxon exhibiting morphological divergence from previously recorded *Rhododendron* species. However, flowering specimens remained elusive until the field survey in May 2025, when flowering individuals were finally observed and their morphology was measured and described *in situ*. Based on comprehensive morphological evidence, we propose that it represents an undescribed species. To ascertain the taxonomic independence of the proposed new species, we also performed phylogenomic analyses based on genome resequencing data. The resulting species tree provided preliminary molecular evidence supporting its recognition as a new taxon.

## Methods

### Materials collection and morphological comparison

In May 2025, flowering individuals were collected from Luohanba in Daguan County. Fresh leaf vouchers were prepared, dehydrated in sealed bags with silica gel, and stored as molecular vouchers. To assess the distribution and population status of the newly discovered taxon, we performed repeated field surveys in its type locality and the surrounding area of the Nature Reserve in Daguan and Yiliang Counties. Additionally, we carried out a comprehensive literature examination (Fang et al. 2005; Geng 2014) and critical examination of relevant herbarium specimens from the online database CVH (http://www.cvh.ac.cn) and the JSTOR Global Plants (http://plants.jstor.org).

### DNA sequencing and phylogenetic analysis

Total genomic DNA of the new species was extracted using the modified CTAB method (Doyle and Doyle 1987). Sequencing libraries were prepared and resequenced in paired-end mode on the DNBSEQ-T7 platform (Wuhan Benagen Co., Ltd.). In addition, whole genome sequencing (WGS) data of six species from *Rhododendron* subsect. *Maculifera* and *Vaccinium oldhamii* (Ericaceae) were retrieved from the Genome Sequence Archive (GSA) database of the NGDC (National Genomics Data Center) and the NCBI (National Center for Biotechnology Information) (Table 1).

**Table 1.** Source of materials studied and accession numbers.

Taxon	Accession ID
*R. pachytrichum*	CRR361043
*R. maculiferum*	CRR360969
*R. ochraceum*	CRR360931
*R. strigillosum*	CRR360971
*R. longesquamatum*	CRR361027
*R. pachyphyllum*	CRR361110
*Vaccinium oldhamii*	SRR29122482

The obtained raw WGS data were first processed with fastp v0.23.4 (Chen 2023) using the default parameters to control quality. Using 5358 single-copy nuclear orthologous genes previously screened from the genus *Rhododendron* (Mo et al. 2025) as references, putative single-copy orthologous genes were assembled from the WGS data with HybPiper v2.3.3 (Johnson et al. 2016) under default parameters. Gene sequences were extracted for each sample using the “retrieve_sequences” module in HybPiper. Sequences were aligned with MAFFT v7.526 using the “auto” option. Gene trees were constructed using IQ-TREE v3.01 (Nguyen et al. 2015) under the “MFP” option with 1000 bootstrap replicates. Newick Utilities (Junier and Zdobnov 2010) were used to prune branches in gene trees with bootstrap values < 30. Species trees were inferred from the resulting gene trees with ASTRAL v1.23.4.6 (Zhang et al. 2018) under default settings. *Vaccinium oldhamii* was selected as the outgroup to re-root the species tree in FigTree v1.4.4 (https://github.com/rambaut/figtree). Finally, the species tree was visualized and displayed using FigTree.

## Results and discussion

**Figure 1.**
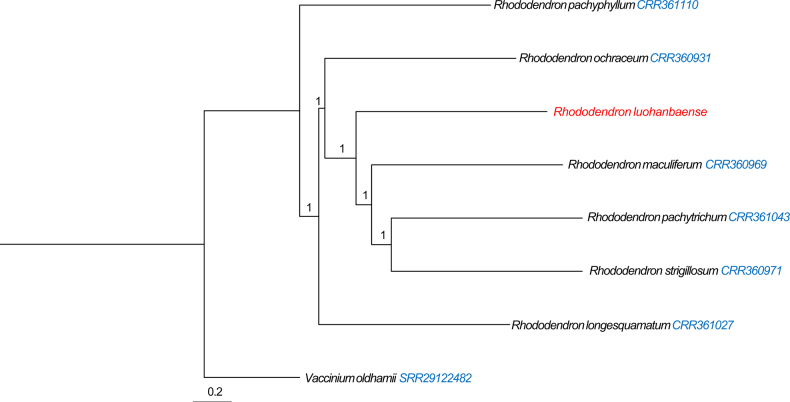
ASTRAL species tree of *Rhododendron luohanbaense* and related taxa. Note: Node values represent local posterior probabilities (LPP).

On molecular basis, after branch pruning, 5357 gene trees were obtained and subsequently used to reconstruct the species tree. The new species formed a stable, fully supported (LPP = 1) clade that was sister to a closely related group comprising *R. maculiferum*, *R. pachytrichum* and *R. strigillosum*. It represents an independent, fully supported (LPP = 1) monophyletic lineage. This phylogenetic placement rules out the possibility that the new species represents a population of either *R. maculiferum* or *R. pachytrichum* (Fig. 1). However, further research based on additional specimens is needed to clarify its exact phylogenetic placement.

### Taxonomic treatment

#### Rhododendron luohanbaense

Taxon classificationPlantaeEricalesEricaceae

Y.L.Zhao & D.T.Liu sp. nov.

3A53454D-3B98-5C35-A504-ABE58E8F4FC6

urn:lsid:ipni.org:names:77379003-1

Figs 2, 3

##### Diagnosis.

**Figure 2.**
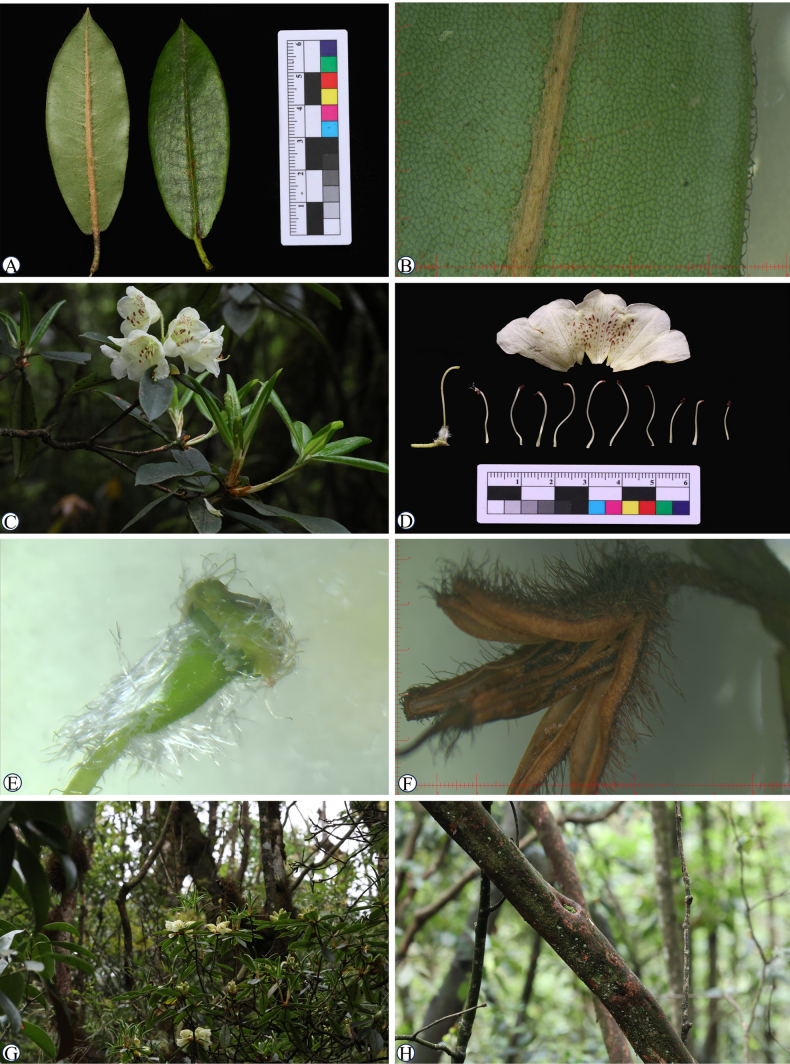
*Rhododendron luohanbaense* Y.L.Zhao & D.T.Liu, sp. nov. **A**. Leaf; **B**. Leaf, abaxial midrib; **C**. Flowering branch; **D**. Dissection of flower; **E**. Ovary; **F**. Opened capsule; **G**. Habitat; **H**. Stem.

**Figure 3.**
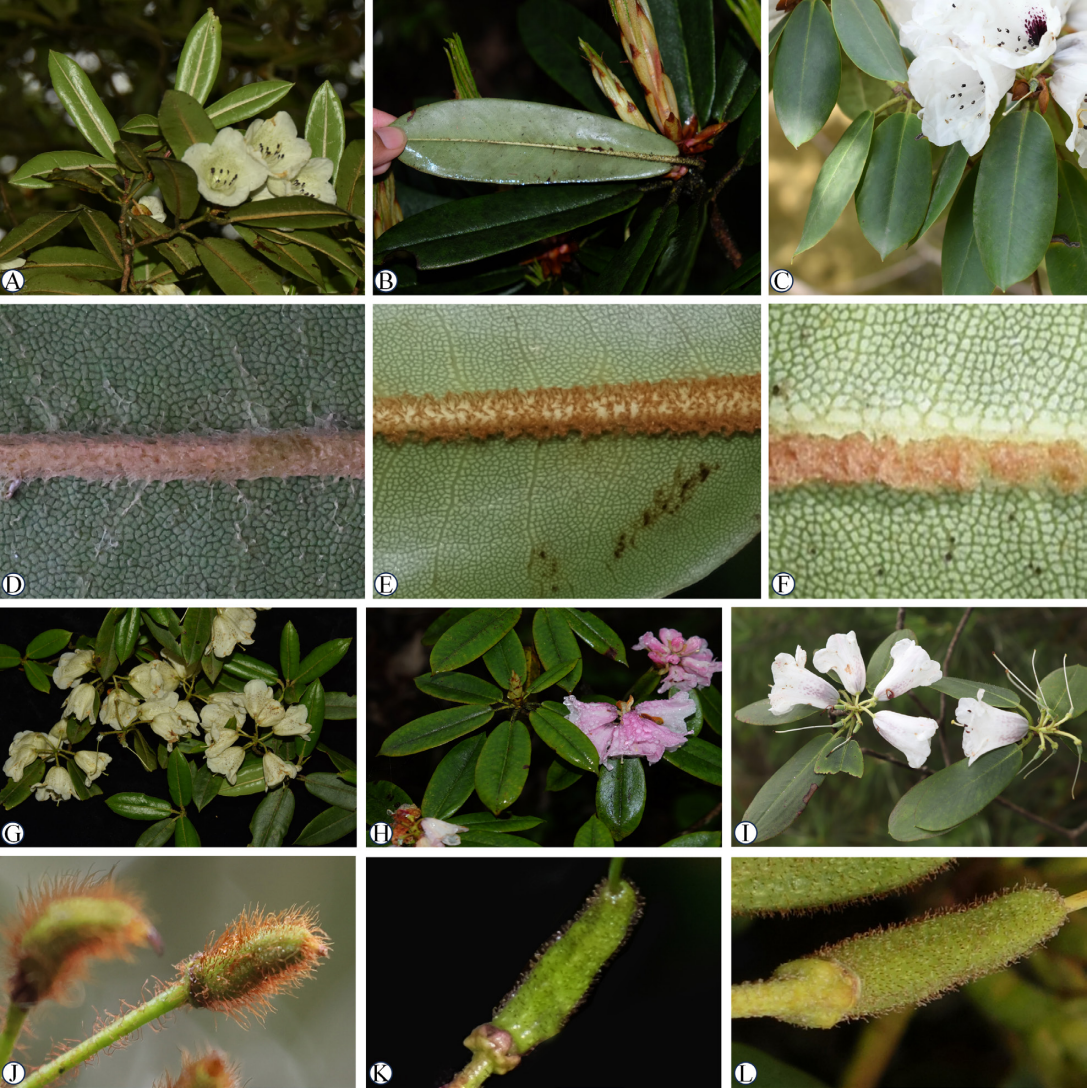
*Rhododendron luohanbaense* (**A, D, G, J**), *Rhododendron pachytrichum* (**B, E, H, K**) and *Rhododendron maculiferum* (**C, F, I, L**). **A–C**. Leafy branches; **D–F**. Abaxial midrib of leaves; **G–I**. Flowering branch; **J–L**. Capsule.

*Rhododendron luohanbaense* resembles *R. pachytrichum*, but can be distinguished by its markedly shorter leaves (4–7 cm vs. 7–14 cm), the densely woolly leaf abaxial midrib (vs. curved floccose-tomentose mainly near base), yellowish-green corollas (vs. white to pink) and the densely long brown or rufous setose capsule (vs. ferruginous-tomentose or glabrescent) (Figs 2, 3, Table 2). The new species is also most similar to *R. maculiferum*, but differs significantly in having markedly narrower leaves (2–3 cm vs. 2.5–4.2 cm) with acuminate apexes (vs. obtuse or rounded apexes), the leaf abaxial midrib entirely covered with dense woolly hairs (vs. densely tomentose under the middle part), yellowish-green to pale green corollas, and 4–7 flowers (vs. 7–10 flowers), as well as a densely long brown or rufous setose capsule (vs. rufous-tomentose or glabrous) (Figs 2, 3, Table 2). More detailed morphological differences among the three similar species are shown in Table 2.

**Table 2.** Comparison of *Rhododendron luohanbaense*, *R. pachytrichum* and *R. maculiferum*.

Characters	*R. luohanbaense*	*R. pachytrichum*	*R. maculiferum*
Leaf size	4–7 × 2–3 cm	7–14 × 2–4.5 cm	4–11 × 2.5–4.2 cm
Leaf apex	acuminate	obtuse to acuminate	obtuse or rounded
Leaf abaxial midrib	densely long woolly from base to apex	curved floccose-tomentose near base	densely tomentose near base
Petiole length	1–1.2 cm	1–1.5 cm	1.4–2.5 cm
Lateral veins	9–12	14–19	12–17
Inflorescence	4–7	7–10	7–10
Corolla shape	campanulate	campanulate	open campanulate
Corolla size	2.2–3.5 × 2.8–4.5 cm	3–4.5 × 3–4.2 cm	3.7–4 × 3.8–4.2 cm
Corolla color	yellowish-green to pale green	white to pink	white to pale pink
Filament	glabrous	white-pilose at base	white-pilose at base
Ovary	densely long-white-pilose	densely rufous-tomentose	slightly pale-brown-tomentose or glabrous
Capsule	densely long brown or rufous setose	ferruginous-tomentose or glabrescent	rufous-tomentose or glabrous
Distribution	NE Yunnan	Sichuan, Chongqing, NE Yunnan, etc.	Sichuan, SW Shaanxi, S Gansu, W Hubei, and Guizhou, etc.
Elevation	1820–2050 m	1700–3500 m	1600–3400 m
Flowering	April–May	April–May	May–June
Fruiting	November	August–September	September–October

##### Type.

China • Yunnan, Zhaotong City, Daguan County, Tianxing Town, Lvnan Village, Luohanba, 27°53'58.16"N, 104° 6'9.87"E, elev. ca. 2048 m, in subtropical montane evergreen broadleaved forest, under forest, 15 May 2025, *Y.L. Zhao*, *D.T. Liu*, *C.S. Gu* & *Z.H. Liao WMS033* (holotype KUN, isotype KUN).

##### Description.

***Shrub or small tree, evergreen***. 1–8 m tall, bark brown, smooth. ***Young branches*** green, densely covered with white tomentose, aging to brown and gradually glabrous. ***Leaves*** subleathery, clustered subverticillately at branch tips; ***petiole*** 1–1.2 cm long, white-tomentose when young, rapidly brown-black tomentose-setose; ***leaf blade*** elliptic-lanceolate to ovate-lanceolate, 4–7 cm long, 2–3 cm wide, base orbicular to cordate, margin ciliate (white when young and black when old), apex acuminate (shortly acuminate); ***adaxial surface*** green, glabrous except at midrib base, grooved; ***abaxial surface*** pale green, midrib prominent, densely covered with long white woolly when young, gray to brown when mature; ***lateral veins*** 9–12 pairs, sparsely brown-woolly. ***Inflorescence*** a terminal short racemose-umbel, 4–7 flowered; ***rachis*** 1–1.8 cm, glabrous; ***pedicels*** green or brown, 1–2.5 cm long, densely white-pilose. ***Calyx*** ca. 1.5 mm, lobes 5, sharply triangular, densely white-pilose. ***Corolla*** campanulate, 2.2–3.5 cm long, 2.8–4.5 cm in diameter, yellowish-green to pale green, internally with yellow-green, brown, purplish-red flecks, lacking or with a basal blotch; ***lobes*** 5, orbicular, 0.7–1.1 cm long, 1.4–1.8 cm wide, apex emarginate. ***Stamens*** 10, unequal, 1–2.8 cm long; ***filaments*** glabrous; anthers oblong, 1–2 mm long, purplish-black. ***Ovary*** narrowly conical, 4–5 mm long, densely covered with long-white-pilose; ***style*** 2–3 cm long, glabrous; ***stigma*** small, greenish or pale yellow, ca. 1 mm wide. ***Capsule*** cylindrical, ca. 1 cm long, diameter 3–4 mm, straight or slightly curved, densely covered with long brown or rufous setose.

##### Phenology.

*Rhododendron luohanbaense* flowers from April to May and fruits in November.

##### Etymology.

The specific epithet derives from the Luohanba, the subzone of the Yunnan Wumengshan National Nature Reserve where the holotype was collected; Chinese mandarin: luó hàn bà dù juān (罗汉坝杜鹃).

##### Distribution and ecology.

**Figure 4.**
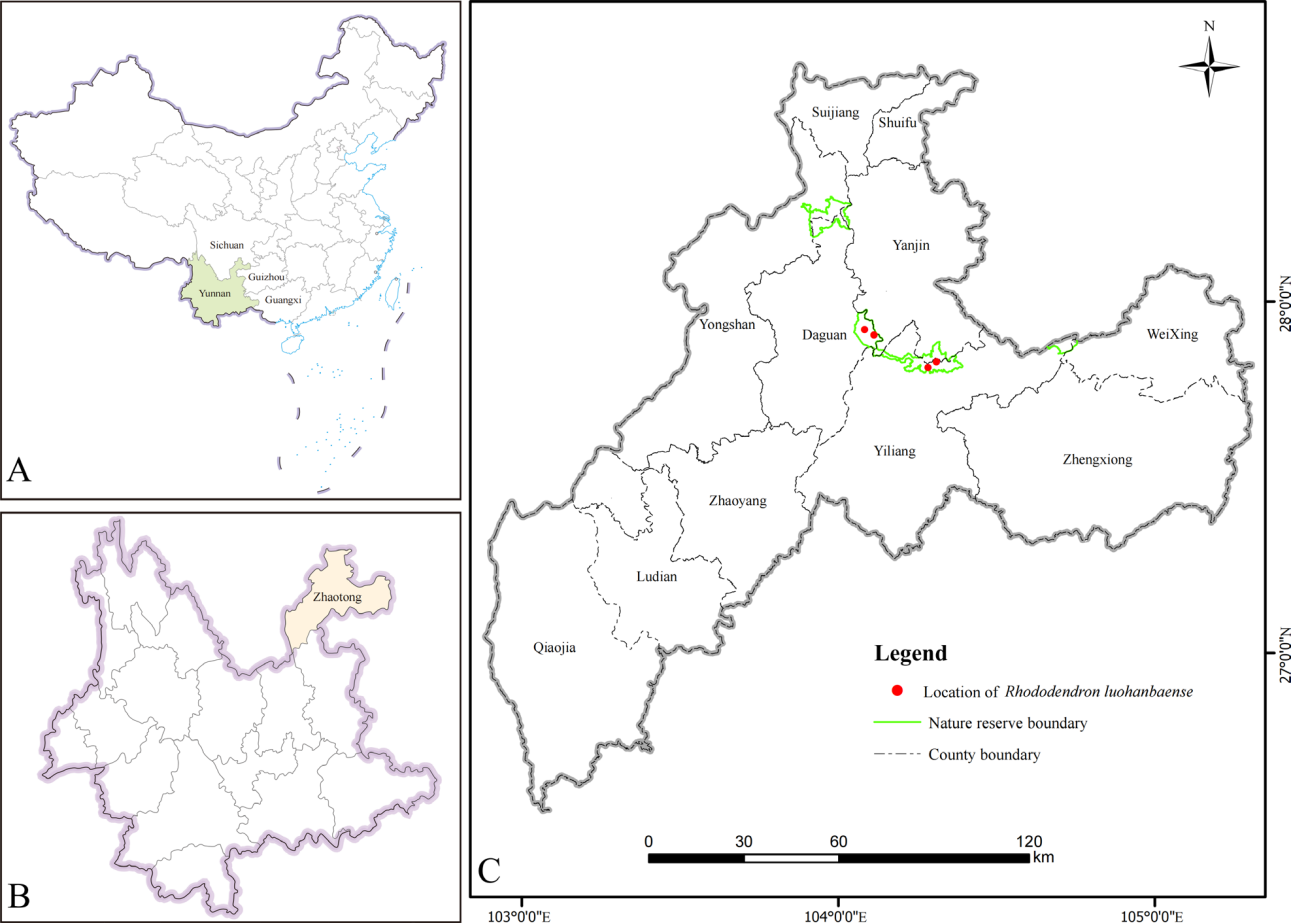
Geographic distribution of *Rhododendron luohanbaense* in Wumengshan National Nature Reserve. **A**. China; **B**. Yunnan Province; **C**. Zhaotong City.

Currently, *Rhododendron luohanbaense* is primarily distributed in Daguan and Yiliang Counties, Zhaotong City, northeastern Yunnan Province, China (Fig. 4). It grows at elevations between 1820 m and 2050 m in subtropical montane evergreen broad-leaved forests and evergreen deciduous broad-leaved mixed forests.

##### Preliminary conservation status.

*Rhododendron luohanbaense* occurs in multiple localities within national nature reserves where human disturbance is limited. Currently, four distribution sites are known in Daguan and Yiliang Counties in protected areas. The estimated extent of occurrence (EOO) is approximately 85 km^2^. Thus, the conservation status is preliminary classified as Endangered (EN) according to the IUCN Red List Categories and Criteria (IUCN 2001).

##### Notes.

Based on gross morphological characters comparison, the plants recently collected from Luohanba belong to subsection *Maculifera* (Fang et al. 2005). This assignment is supported by the diagnostic features of this subsection: abaxial leaf indumentum denser along the midrib, ovary densely pilose, and campanulate corolla internally with flecks or blotch. Within this subsection, the new species is most similar to *R. pachytrichum* and *R. maculiferum* in sharing denser indumentum along the abaxial leaf midrib, sparse inflorescence, and campanulate corolla (Fig. 3).

Except for the difference in the Diagnosis section. The three species show clear divergence in distribution, elevational range, and phenology. *R. luohanbaense* is currently known only from NE Yunnan at mid-elevations (1820–2050 m). In contrast, *R. pachytrichum* occupies a broader elevational range (1700–3500 m) across Sichuan, Chongqing, Shaanxi. *R. maculiferum* has a wide distribution spanning Shaanxi, Gansu, Hubei, Sichuan, Guizhou.

##### Additional specimens examined

**(*paratype*)**. China • Yunnan, Zhaotong City, Yiliang County, Xiacaoba Town, Houhe, 27°49'27.2847"N, 104°18'08.9662"E, elev. ca. 1923 m, in subtropical evergreen broad-leaved forest, under forest along valley, 19 May 2025, *Y.L. Zhao*, *D.T. Liu*, *C.S. Gu WMS053* (KUN).

## Supplementary Material

XML Treatment for Rhododendron luohanbaense

## Additional information

### Use of AI

No use of AI was reported.

### Author ORCIDs

Yong-Li Zhao https://orcid.org/0009-0000-7526-4375

Chao-Shan Gu https://orcid.org/0009-0005-0788-8705

Zheng-Hang Liao https://orcid.org/0009-0004-0678-8297

Wei Li https://orcid.org/0009-0001-8976-3842

Zhu-Fang Xie https://orcid.org/0009-0001-3705-5094

Fei Wang https://orcid.org/0009-0004-1249-2193

Yong-Peng Ma https://orcid.org/0000-0002-7725-3677

Xing-Xing Mao https://orcid.org/0009-0006-2313-8004

De-Tuan Liu https://orcid.org/0000-0002-2295-3799

### Data availability

All the sequencing data used in this study are publicly available in the National Genomics Data Center (NGDC) and the National Center for Biotechnology Information (NCBI). The resequencing data for *Rhododendron luohanbaenseare* was held in reserve for a related study.

## References

[B1] Chang YH, Yao G, Neilsen J, Wang SH, Ma YP (2021) *Rhododendron kuomeianum* (Ericaceae), a new species from northeastern Yunnan (China), based on morphological and genomic data. Plant Diversity 43(4): 292–298. 10.1016/j.pld.2021.04.003 PMC8390914 34485771

[B2] Chen SF (2023) Ultrafast one-pass FASTQ data preprocessing, quality control, and deduplication using fastp. iMeta 2(2): e107. 10.1002/imt2.107 PMC10989850 38868435

[B3] Deng YH, Pan B, Ding T, Liu H, Qin XL, Huang ZX, Deng MM (2025) *Rhododendron yuanbaoshanense* (Ericaceae), a new species from Guangxi, China. Phytotaxa 682(2): 179–184. 10.11646/phytotaxa.682.2.6

[B4] Doyle JJ, Doyle JL (1987) A rapid DNA isolation procedure for small quantities of fresh leaf tissue. Phytochemical Bulletin 19: 11–15.

[B5] Fang MY, Fang RZ, He MY (2005) Flora of China. VOL. 14. Science Press, Beijing, Missouri Botanical Garden Press, St. Louis, 242–517.

[B6] Geng YY (2014) The genus *Rhododendron* of China. Shanghai Scientific and Technical Publishers, Shanghai, 1–612.

[B7] IUCN (2001) IUCN Red List categories and criteria. IUCN Species Survival Commission.

[B8] Johnson MG, Gardner EM, Liu Y, Medina R, Goffinet B, Shaw AJ, Zerega NJC, Wickett NJ (2016) Hybpiper: extracting coding sequence and introns for phylogenetics from high-throughput sequencing reads using target enrichment. Application in Plant Science 4(7): 1600016. 10.3732/apps.1600016 PMC4948903 27437175

[B9] Junier T, Zdobnov EM (2010) The Newick Utilities: High-throughput phylogenetic tree processing in the UNIX shell. Bioinformatics 26(13): 1669–1670. 10.1093/bioinformatics/btq243 PMC2887050 20472542

[B10] Linnaeus C (1753) Species plantarum, exhibentes plantas rite cognitas, ad genera relatas, cum differentiis specificis, nominibus trivialibus, synonymis selectis, locis natalibus, secundum systema sexuale digestas. Laurentii Salvii, Stockholm, 392–393. 10.5962/bhl.title.59734

[B11] Mo ZQ, Fu CN, Twyford AD, Hollingsworth PM, Zhang T, Yang JB, Li DZ, Gao LM (2025) Evaluating the utility of deep genome skimming for phylogenomic analyses: A case study in the species-rich genus *Rhododendron*. Plant Diversity 47(2025): 593–603. 10.1016/j.pld.2025.04.006 PMC12302500 40734829

[B12] Nguyen LT, Schmidt HA, Haeseler AV, Minh BQ (2015) IQ-TREE: a fast and effective stochastic algorithm for estimating maximum-likelihood phylogenies. Molecular Biology and Evolution 32: 268–274. 10.1093/molbev/msu300 PMC4271533 25371430

[B13] Tian XL, Chang YH, Neilsen J, Wang SH, Ma YP (2019) A new species of *Rhododendron* (Ericaceae) from northeastern Yunnan, China. Phytotaxa 395(2): 66–70. 10.11646/phytotaxa.395.2.2

[B14] Zhao YL, Li FQ, Liu ED, Li W (2024) Ecological niche and interspecific association of species of *Cercidiphyllum japonicum* community in Yunnan Wumeng Mountain National Nature Reserve. Hunan Forestry Science & Technology 51(2): 33–34.

[B15] Zhang C, Rabiee M, Sayyari E, Mirarab S (2018) ASTRAL-III: polynomial time species tree reconstruction from partially resolved gene trees. BMC Bioinformatics 19: 153. 10.1186/s12859-018-2129-y PMC5998893 29745866

[B16] Zhao YL, Yi XB, Hao YS, Chen T, Ma YP, Liu DT (2025) *Rhododendron wumengense* sp. nov. (Ericaceae) from northeast Yunnan, China. Phytotaxa 732(2): 212–216. 10.11646/phytotaxa.732.2.8

